# Air Scholars program: A framework for empowering future generations to address climate change

**DOI:** 10.1016/j.isci.2023.108776

**Published:** 2024-01-23

**Authors:** Mahlet Garedew, Jesse John, Alifa Alam, Anthony Buchfuhrer, Leah Dasilva, Fatima Hashem, Kianni Vestal, Courtney Jiggetts, Grant Pace, Carolyn Kissane, Neva Luthria, Tatiana Bravo, John Baker, Gregory Constantine, Stafford W. Sheehan

**Affiliations:** 1 Air Company, 407 Johnson Avenue, Brooklyn, NY 11206, USA; 2 NYU Center for Global Affairs, 15 Barclay Street, New York, NY 10007, USA

**Figure:**
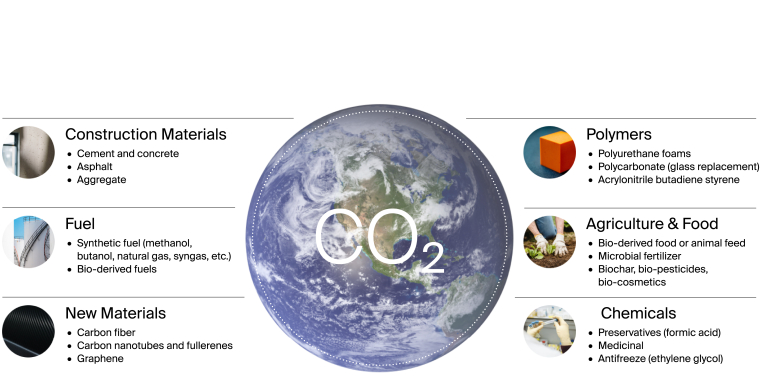
Above image: This figure illustrates some of the value-added products that can be produced through CCUS technology, including sustainable fuels, carbon-negative plastics, carbon-negative building materials, etc. These products can help to reduce greenhouse gas emissions and promote sustainable development across a range of sectors. The figure highlights the potential benefits of CCUS beyond emissions reduction and underscores the importance of exploring these opportunities to support the transition to a low-carbon economy.

“The program heightened my awareness of the repercussions of climate change on marginalized communities globally.”

“I am now more motivated to advocate for climate justice and to support innovative solutions like CCUS to benefit the most vulnerable populations.”

“The fact that I could showcase my creativity to make a greater difference in underrepresented communities ultimately influenced me to change my trajectory in terms of future career paths and I’m excited for what the future holds.”

“This is not just about technological deployment; it is about ushering in a new era of environmental justice, grounded in safety, empowerment, and equity.”

“The program underscored that climate change isn't just a matter of science or politics—it's deeply personal and ethical.”

“The Air Scholars program has taught me that climate change is not just an environmental issue, but also a social justice issue that disproportionately affects communities of color.”

“The students’ potential was evident in their eagerness to learn about the CCUS space and in the nuanced questions they asked.”

“Most importantly, they understood their active agency to engage in this important work area.”

## Main text

Greenhouse gases (GHGs) added to the atmosphere today pose a greater burden on future generations than present ones. Fossil fuels are the incumbent carbon and energy source for modern life, and action must be taken to transition our fossil fuel-based economy to a sustainable one. Technologies to effect change in this manner already exist, but to deploy them in a way that is best for humanity and to mitigate climate change, education is a critical factor. Awareness for climate change has broadly increased in recent years, as education in schools around climate change, GHG emissions, and carbon footprints has improved. However, understanding of what we can do with atmospheric carbon dioxide to displace the need for fossil fuels or permanently remove carbon is still needed for society to come together and address climate change. The methods that future generations will need to use on a massive scale to fight climate change, such as carbon dioxide utilization, are not typically digested into subjects understandable by the non-technical public or, more importantly, taught in school.

As a carbon dioxide capture and utilization technology developer in New York City, Air Company has a unique opportunity and responsibility to further climate change education for New Yorkers. The first (and perhaps most logical) place to contribute to the education of students in formative years was a high school. Thus in 2022, the Air Scholars program was launched in partnership with the Brooklyn Academy of Science & the Environment (B.A.S.E.). High school students sit at a unique intersection of cognitive readiness, curricular flexibility, and formative decision-making, making them an optimal pilot group for the first Air Scholars cohort. The program teaches students about the underlying causes of climate change and how civilization can address it at large scale, empowering them to become advocates and responsible stewards of the environment. By fostering direct interaction through structured discussions and mentorship initiatives, Air Scholars also aims to demonstrate to students the diverse pathways available to them within the climate field, spanning not only science, technology, engineering, and mathematics (STEM) but also other disciplines, like design, entrepreneurship, and communications.

This backstory delves into the program’s framework, motivations, and outcomes, while spotlighting the invaluable perspectives of those involved. Through the voices of participating students, faculty collaborators, company employees, program coordinators, teachers, and mentors, this paper seeks to showcase the effectiveness of a collaborative educational framework that successfully integrates industry insights with pedagogy to create a profound impact on student learning.

### Educational immersion: Sparking intellectual curiosity and engagement

One of the core pillars of the program is an immersive educational experience, designed to engage students in literature review, structured discussions, and writing sessions. These activities fostered dialogue not only among student peers but also with the program supervisor/teacher, faculty advisor, and program coordinators, providing a nurturing environment for students to learn and grow. The program further enriched students' learning and insights through departmental presentations and site visits, providing glimpses into CO_2_ conversion technology and different departments in the business. In this section, the pivotal role of Dr. Jesse John, Student Research Supervisor who curated the pedagogical framework for the Air Scholars program, is underscored. Additionally, the depth of student learning is shown by their responses to selected key questions tackled during the the program’s duration.

#### Pedagogical framework

The Air Scholars program was an incredibly ambitious endeavor that was centered around students being fully immersed in the scientific literature on environmental justice and carbon capture, utilization, and storage (CCUS). It was imperative for us to develop a multifaceted program that would fully support students in distilling the technical jargon, complex data, and nuance that accompanies scientific literature using the TRU (teaching for robust understanding)^1^^,^^2^ framework and the C.R.E.A.T.E (Consider, Read, Elucidate the hypotheses, Analyze and interpret the data, and Think of the next Experiment) method.^3^ These tools provided the necessary scaffolding for scholars to evaluate the quality of the research and contextualize the findings within the broader scientific landscape. The dimensions of this framework offer high-quality STEM opportunities for students to emerge as knowledgeable, resourceful thinkers, and problem solvers. Using the TRU framework as a compass, we designed learning experiences where students are required to grapple with questions that trigger curiosity and prolong student engagement. These lessons include open-ended questions such as, “What steps are being taken to ensure that CCUS or carbon dioxide removal (CDR) technologies are safe for local communities in the United States?” Such cognitive tasks allow students to learn about sustainability and curate both problem-solving and critical thinking skills, which require students to productively struggle.

One of the major challenges and accomplishments of the Air Scholars program was its focus on reading and analyzing scientific literature. A modified version of the C.R.E.A.T.E method to scaffold the scientific literature was utilized.^3^ The students were encouraged to ask questions and share their own perspectives on various aspects of the climate crisis. Each learning module began with students reading distilled versions of the target research papers. These sources offered an appropriate scaffold that was engaging, informative, and accessible. Once students had a firm grasp on the main concepts, the target publication would be introduced. Students engaged in activities such as concept mapping the introduction, deciphering unfamiliar vocabulary, creating box models for experimental sections, weekly meetings with experts in the field, and designing subsequent experiments. Over time, these exercises honed their critical thinking and confidence, enabling them to independently interpret and delve into the scientific literature and eventually propose their own potential solutions to climate-related challenges, positioning them as informed and proactive contributors to the global dialogue on environmental sustainability.

Throughout the program, the Air Scholars were engaged in a variety of activities that helped them contend with the complexities of the global climate crisis and the role CCUS technology can play in addressing it. The program provided lab experiences, professional mentorship, expert panel discussions, and opportunities to present their research to variable audiences. This structure allowed the scholars to grow professionally as they explored real-world solutions and cutting-edge technologies that can impact global climate change. As the program progressed, students felt empowered to voice their nuanced opinion on the intersection of climate issues, environmental justice, and CCUS technology. They became more aware of the scale of the anthropogenic impact on the environment and more confident in their own abilities to make a difference. The students were excited about the possibilities of CCUS and saw a future where their personal career paths could directly or indirectly impact the imminent climate crisis.

#### Student learnings and reflections

Throughout the Air Scholars program, students reflected on their personal career paths and interests (captured in Question 1) and their perspectives on climate solutions (captured in Question 2) as well as delved into numerous case studies, examining the ramifications of deploying technologies in diverse communities globally.

### Question #1: What are your future career interests? Can you envision yourself working on CCUS?

**Collective** r**esponse from Air** **Scholars:** Through our involvement in the Air Scholars program, we’ve recognized the urgent necessity to combat climate change and the pivotal role CCUS technologies have in this challenge. As we delved deeper into the mission of climate change organizations and the impact of CCUS, our interest in such careers grew. This program has reshaped our career paths, highlighting that every industry has the potential to address climate change. Inspired by this, we are steering our future careers toward innovations and sustainable solutions for the climate crisis. Below are our individual reflections on our career aspirations.

**Fatima:** Originally aspiring to be a pediatrician, my focus shifted toward environmental science after understanding the implications of CDR technology and the disproportionate effects of climate change on low-income families. I aim to work on CCUS solutions that benefit these communities through sustainable energy and job creation.

**Kianni:** While I always wanted to be a fashion designer, the program introduced me to the potential of merging my passion with climate solutions. By using materials derived from CCUS processes, I can contribute to reducing GHG emissions.

**Anthony:** The diverse career opportunities in the climate industry became clear to me through the Air Scholars program. Beyond the technical side, there’s a need for marketing, operations, and education to maximize the impact of CCUS technologies.

**Alifa:** With a keen interest in marketing, I see myself promoting CCUS solutions globally through various media channels.

**Leah:** The program illuminated the links between environmental justice and the climate crisis, showing me the avenues through which I can advocate for disproportionately affected communities.

### Question #2: How has the program changed your perspective on the impact and solutions of climate change?

**Collective response from AirScholars:** Our understanding of climate solutions expanded beyond traditional methods like reforestation after joining the Air Scholars program. The potential of CDR and CCUS technologies became clear, and the program equipped us to understand and convey key climate change mitigation strategies.

**Fatima:** The Air Scholars program has taught me that climate change is not just an environmental issue but also a social justice issue that disproportionately affects communities of color.

**Kianni:** Before joining the Air Scholars program, I didn’t realize the extent of the environmental racism and injustice that exists in our communities. The program has opened my eyes to the fact that climate change is not a problem that can be solved by individuals alone but rather requires systemic change and collective action. I am now more motivated to advocate for climate justice and to support innovative solutions like CCUS to benefit the most vulnerable populations.

**Anthony:** Learning about CCUS technologies has shown me that there is a wide portfolio of solutions available that can help reduce greenhouse gas emissions and make a real impact in the fight against climate change.

**Alifa:** The program heightened my awareness of the repercussions of climate change on marginalized communities globally.

**Leah:** The program underscored that climate change isn’t just a matter of science or politics—it’s deeply personal and ethical.

#### Final class activity and student reflection

As a pivotal culminating activity, students stepped into the shoes of community stakeholders, specifically in a CCUS project scenario. Tasked with the challenge of identifying measures to safely introduce CCUS technologies into their own communities, students showcased their understanding of the topic as they each honed in on a specific area, crafting guiding principles to apply in the future. Their focuses spanned from the necessity of legally binding contracts,^4^ ensuring equitable benefits for all,^5^ and devising risk prevention and recovery plans,^6^ to the importance of financial and technical assistance,^7^ and the role of government oversight^8^ in the deployment of these climate solutions.

**Collective reflection:** Our time in the Air Scholars program brought to light the disproportionate effects of climate change on communities of color. These communities often bear the brunt of environmental consequences from industrial actions, further compounded by economic challenges. It’s vital for CCUS companies to conscientiously integrate their technologies with these communities. This is not just about technological deployment; it is about ushering in a new era of environmental justice, grounded in safety, empowerment, and equity.

#### Continued empowerment: From summer insights to year-round development

The culmination of the six-week summer program was marked by students presenting their learnings and findings to company employees. This platform allowed them to showcase their growth, confidence, and newfound knowledge, affirming the transformative impact of the program. To ensure sustained learning and personal development, an Air Scholars after-school program was implemented throughout the academic year. The extension of the program into the school year helped nurture critical thinking and writing skills, providing students with an ideal space to continue their educational journey.

#### Student Research Supervisor and faculty advisor reflections

**Dr. Jesse John**, a geochemist from Stony Brook, actively applies his expertise in synthesizing novel materials and sustainable solutions in his role at the High School for Innovation in Advertising and Media. Through his mentorship, students have showcased their work on sustainable solutions at notable conferences. In addition to teaching, he founded Explainables, a nonprofit focused on making scientific discussions more accessible to the public. Dr. John serves as the Air Scholars Research Supervisor.

**Jesse:** Participating in the Air Scholars program was deeply rewarding. Witnessing the potential of these students reaffirmed my commitment to nurturing the next generation of environmental advocates. Their zeal inspires me daily, emphasizing the significance of CCUS in addressing our global climate crisis.

**Dr. Carolyn Kissane**, an Academic Director at New York University, brings a wealth of knowledge from energy geopolitics to climate change and security. Her roles extend to directing the SPS ECJS (School of Professional Studies Energy, Climate Justice, and Sustainability) Lab and serving as faculty adviser to the Energy Policy International Club. She collaborates closely with the Air Scholars, guiding them through in-depth discussions on environmental literature.

**Carolyn:** Working with the Air Scholars was an honor and privilege. It was fantastic to work with scholars as they took the time to examine environmental and climate justice issues and carbon capture and conversion technologies and applications. Students read the latest literature and applied their knowledge in weekly discussions and conversational deep dives, asking questions about applications and impacts on vulnerable populations. Our weekly meetings provided the space and opportunity to deconstruct and look at environmental justice from different perspectives. Environmental justice requires careful consideration around the fair treatment and authentic involvement of all people, irrespective of race, color, national origin, or income, concerning developing, implementing, and enforcing environmental laws, regulations, and policies. During the summer of 2022, Air Scholars asked questions about the degree of protection around carbon capture and conversion technologies and examined health hazards, locational proposals, and impacts on carbon mitigation. Most importantly, they understood their active agency to engage in this important work area. Throughout the summer over 6 weeks, students had the opportunity to do site visits, talk with experts in carbon capture and conversion applications, and work together to develop an intellectual and practical foundation in climate change and solutions. It was a fantastic pleasure and opportunity to work with them in my capacity as an educator and intellectual guide.

### College preparation: Paving the path to success

Air Scholars is multifaceted in its approach to providing our student cohorts with access to personal, professional, and academic growth opportunities. Working in tandem with tailored literature review and engagement encompassing subjects such as climate change, CCUS, and environmental justice, we offer the cohort optional college prep and Scholastic Aptitude Test (SAT) sessions to help further guide them on their post-graduation journeys. The college prep sessions cover a breadth of topics, ranging from scholarships to resume/cover letters, and we tap external professionals for additional insight as needed (e.g., students may hear from interviewers of specific scholarships they are applying for). This portion of the summer curriculum prioritizes the college essay/personal statement that students will have to write when applying for their desired undergraduate programs. In both group and one-on-one sessions, our scholars are assisted in brainstorming as well as editing any existing essay drafts they may have. Generally, the college prep sessions introduce students to subject matter to help guide them in their next chapter, but the structure remains malleable to accommodate student needs/specific interests. The SAT sessions gave students the opportunity to review and study resources for SAT Math and English content, take several practice tests, and review missed questions in both group and one-on-one sessions. Through this setup, students were able to both teach and be taught by one another through their participation. Students’ results were converted into corresponding scores and measured over time, giving them the opportunity to see measurable improvement as the result of their work and dedication.

#### Program founder/coordinator reflections

Courtney Jiggetts (Environmental Strategist) and Grant Pace (Environmental Strategist) were the Air Company team members who conceptualized and founded the program internally. As Environmental Strategists, Courtney and Grant offer support in a number of areas and also ideate and execute initiatives promoting sustainability and environmental justice across Air Company operations. They serve as coordinators of the program and engaged with students as mentors, advisors, as well as SAT and college preparatory coaches. They reflected on what the program meant to them, what inspired them, and what they learned from launching and running this program.

**Program inspiration:** The primary inspiration for the Air Scholars program was the National Aeronautics and Space Administration (NASA)’s Climate Change Research Initiative (CCRI; https://www.giss.nasa.gov/edu/ccri/), a cross-generational internship program that enables high school students, college students, graduate students, and NASA climatologists who serve as experts in their field to work collaboratively in teams focused on specific frontiers in climate research. CCRI aims to produce peer-reviewed publications and give the future generations of climate leaders indispensable training and skills; these goals serve as the pillars for Air Scholars. The Air Scholars program was also partially inspired by larger tech companies’ paid youth mentorship and professional development opportunities for local college-bound high school students, such as Microsoft’s Discovery Program.

**Figure:**
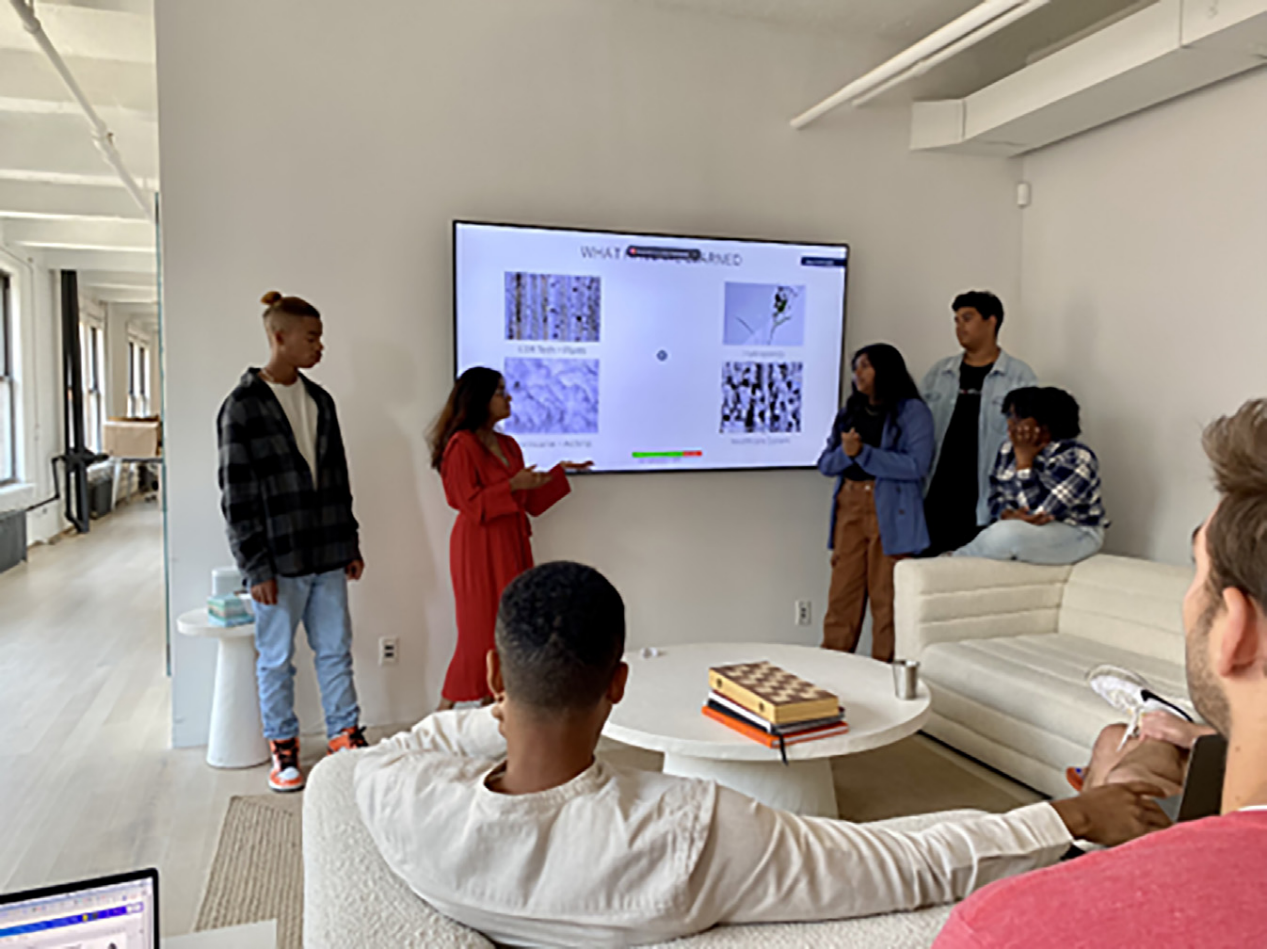
Students presenting their work to company employees on the final day of the program

**Learnings and takeaways:** There were considerable key learnings while facilitating the first Air Scholars pilot cohort that will drive its success moving forward. When building the students’ schedule, time was allocated for them and their Research Supervisor to dissect peer-reviewed research at a consistent pace, but the learning curve involved in teaching high school students how to approach complex materials also needed to be considered. Creating a strategic framework with benchmarks to track student progress and increasing capabilities to engage with these readings will, therefore, be foundational at the commencement of the program in the future. Further, leveraging available local resources from NYU Urban Future Lab and New York State Energy Research and Development Authority (NYSERDA) was essential to the program’s success, demonstrating the potential for creative use of policy-based climate funding for innovative solutions.

**Courtney:** To me, the Air Scholars program is a tight ecosystem that represents what the world *can* be and who all of our systems and industries should serve. Working with these students has been an incredible experience and has demonstrated the beautiful, unyielding commitment that younger generations have to restore our planet and innovate across the systems that have perpetuated climate change and its burdens.

**Grant:** The Air Scholars program is a microcosm representing the kinds of community-based climate solutions needed at large scale to achieve warming targets and ensure environmental justice. Initially, realizing how ambitious we were with our expected student learnings was daunting much like the climate crisis itself; by the end of the program, I was blown away by the powerful dedication and growth of our students, leaving me with more inspiration and climate optimism than ever.

### Career exploration and development: Nurturing ambitions

At the program kickoff event, students had the opportunity to learn about various aspects of the company (e.g., technology, operations, design, marketing, etc.) from presentations by representatives from each department. During the program students also toured different Air Company facilities to learn more about CO_2_ conversion processes at different scales. Aligning with their career aspirations, students were then paired with mentors from the company, creating an opportunity for career exploration and development. This element of the program had a semi-structured format where mentors were provided with recommended discussion topics to guide student engagement. However, the mentor/student pair retained the flexibility to shape and structure their sessions according to the students’ needs and interests. During these weekly interactions, students had the opportunity to discuss career paths, shadow their mentors in person, discuss relevant literature, etc. These mentoring relationships provided invaluable opportunities for students to engage in discussions about the diverse range of career prospects within the CCUS field. Additionally, these interactions also afforded company employees the opportunity to connect with students and share their enthusiasm and insights. Mentors shared their reflection on their involvement in the program and what it meant to them. Neva Luthria (R&D Chemist), Tatiana Bravo (Brand Manager), and John Baker (Marketing Director) are three of the six Air Company employees that served as mentors for the Air Scholars program.

**Figure:**
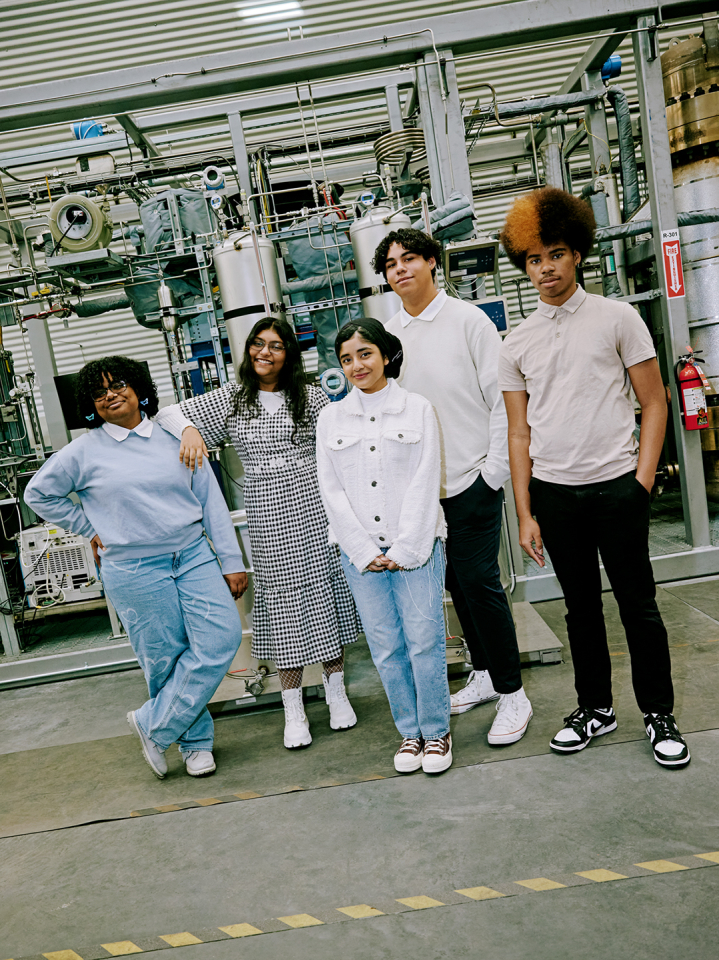
Students touring different Air Company facilities during a plant shutdown (all facility tours were conducted under the supervision of company health and safety personnel)

#### Mentor reflections

**Neva:** Being a part of the Air Scholars mentorship program meant reflecting on my own career trajectory thus far and considering what information or guidance I wish I had known along my journey. The opportunity to pass that along to a younger group of budding scientists and environmentalists was really fulfilling, made especially so by the fact that I got to speak candidly about my experience as a woman in STEM and was able to mentor another young woman on navigating the realities of that path. The students’ potential was evident in their eagerness to learn about the CCUS space and in the nuanced questions they asked. The students came into the Air Scholars program with little to no knowledge about CCUS and finished the program being able to confidently discuss both problems and solutions regarding the role of CCUS in facing the climate crisis and climate injustice. I feel that the Air Scholars program was an impactful way to ground Air Company in its founding values and instill those values in a younger generation who will certainly make a positive impact in innumerable ways with the skills they practiced during the summer.

**Figure:**
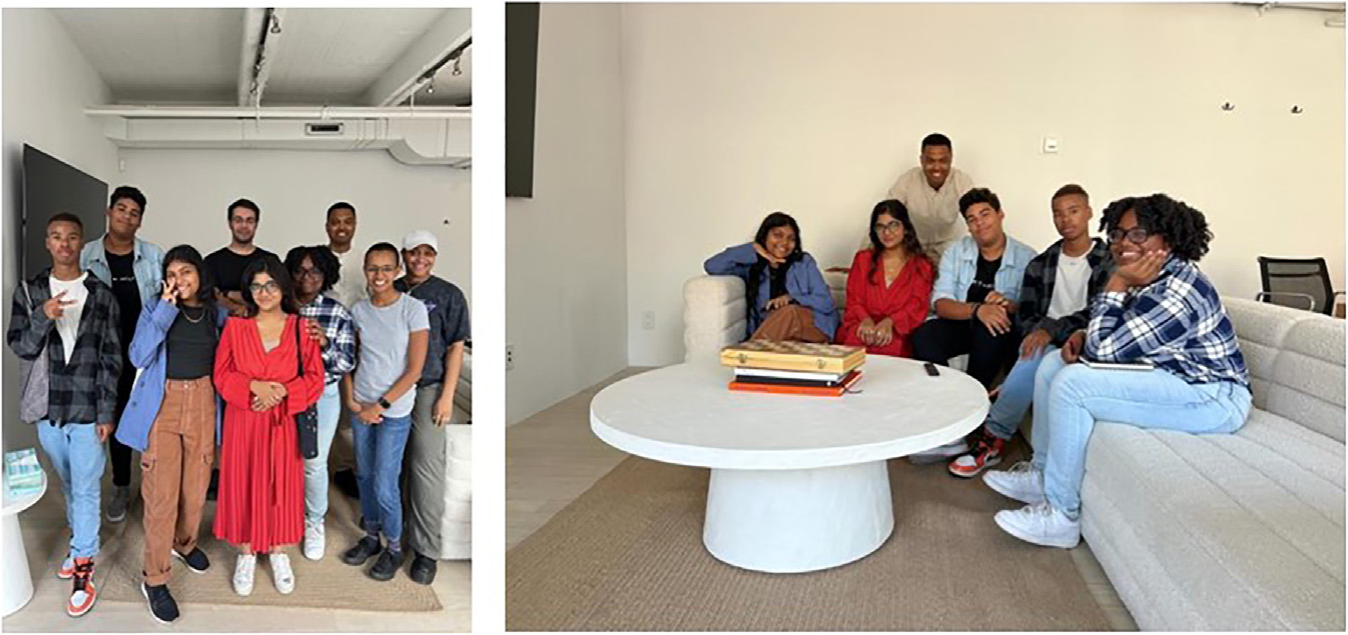
The 2022 Air Scholars cohort along with Program Coordinators and Student Supervisor From left to right (in right photo) Fatima Hashem, Alifa Alam, Jesse John, Anthony Buchfuhrer, Kianni Vestal, and Leah Dasilva.

**Tatiana**: Being a mentor has been one of the most rewarding initiatives I have been a part of at Air Company. During our time together my mentee and I established open communication, allowing her to ask any questions she may have for me about my career or perspective while also teaching me about her life and her goals. We worked through her college admission essay, and I was inspired by my mentee’s perseverance and life outlook. This mentorship program is incredibly valuable as it provides us with the opportunity to empower future generations (particularly women) to set high goals for themselves, and the resources and skills to kick-start their future. The weekly 1-hour meetings with my mentee were often my favorite, and I look forward to watching her take on the world.

**John:** When I met my mentee, I was immediately impressed by the level of drive and focus he had on his education and potential career path. As a B.A.S.E. student, he possessed a keen aptitude for the sciences, but where we really connected was on the subject of creativity. Our weekly touch bases quickly became one of the highlights of my week. Sometimes our conversations centered around the internship experience, and other times we simply talked about life and things we were passionate about outside of work and school. Soon after the internship ended, I learned that my mentee got accepted to the college of his choice on a full-ride scholarship. I couldn’t have been more excited for him, and I have no doubt he’ll go on to do great things.

### Conclusions

The significance of climate change education cannot be underscored enough, especially considering the grave challenges that lie ahead for future generations. The Air Scholars program is a blueprint for other CCUS companies and local communities to replicate. Through the comprehensive pedagogical framework discussed here, and an unwavering focus on student learning and development, the program has shown that it is possible not only to make complex subjects such as CCUS accessible to high school students but also to inspire them to potentially consider careers in this crucial field.

Student reflections from the program paint a vivid picture of empowerment, a newfound appreciation for the intersection of environmental justice, the potential of CCUS technologies, and the overall portfolio of climate solutions. This empowerment was not a by-product of only knowledge transfer but stemmed from active engagement, cognitive challenges, and opportunities for meaningful dialogue. A particularly noteworthy accomplishment of the Air Scholars program was its ability to bridge the gap between advanced scientific literature and high school education. Through scaffolded learning techniques, such as the C.R.E.A.T.E method, students were empowered to grapple with complex topics, further underscoring the importance of adaptive pedagogical techniques in achieving genuine understanding. By reading and analyzing scientific articles, engaging in structured discussions, and receiving mentorship, students were empowered to be active contributors to the discourse on climate change solutions.

Beyond the specifics of the program’s structure and accomplishments, the broader message is clear: there is a dire need to invest in initiatives that educate the youth not only about the realities of climate change but also about the tangible solutions available. It is through endeavors like the Air Scholars program that students can envision themselves as not just witnesses but active participants in the global fight against climate change. As society continues to grapple with the consequences of environmental degradation and seeks viable paths forward, it will be these informed and passionate individuals who will take the mantle, bringing with them the skills, knowledge, and perspectives nurtured in such programs
